# Myths, need, contribution to the diagnosis

**DOI:** 10.1186/1129-2377-16-S1-A5

**Published:** 2015-09-28

**Authors:** Alessandro Bozzao

**Affiliations:** Dipartimento NESMOS, Università Sapienza Roma, Azienda Ospedaliera Sant'Andrea, Rome, Italy

The use of diagnostic imaging in the evaluation of patients with headache is an almost mandatory step. Although there are specific conditions (the so-called red flags) indicating the need of diagnostic imaging, daily experience shows that a study of the brain and, often, of the intracranial vessels, is part of the routine of many of these patients. The most common and appropriate study is the MRI integrated with the angiographic evaluation (MRA). An MRI and MRA, properly performed, are able to exclude most of the organic pathologies potentially causing headache. The list of these conditions is long and not useful in this context. It is important to remember that significant MRI changes are found in about 4% of patients with chronic headache but this percentage can rise up to 14% in the atypical forms. The MRI evaluation also allows to define whether the patients, especially the young ones, have a condition of brain signal alterations (multiple bright spots). Some studies reported that these are more frequent in patients with migraine especially with aura and mostly in females. The clinical significance and role of these changes remains to be proven. Another approach of imaging is related to the possibility of assessing, by means of functional studies, the pathophysiological moments responsible for the symptoms. These studies are based mainly on functional MRI which includes cerebral perfusion, cortical activation (induced or by means of resting state techniques) and metabolic studies with spectroscopy. The last approach is the one that assesses the consequences of long standing symptom analyzing morpho-volumetric brain “in toto” or inside the cerebral cortex. These are techniques like computed voxel based morphometry (VBM) or Free Surfer able to evaluate small volumetric changes between groups of healthy subjects and patients.

